# Considering the influence of land use/land cover on estuarine biotic richness with Bayesian hierarchical models

**DOI:** 10.1002/eap.2675

**Published:** 2022-07-14

**Authors:** Andrew Challen Shamaskin, Sandra B. Correa, Garrett M. Street, Anna C. Linhoss, Kristine O. Evans

**Affiliations:** ^1^ Department of Wildlife, Fisheries, and Aquaculture Mississippi State University Mississippi State Mississippi USA; ^2^ Department of Agricultural and Biological Engineering Mississippi State University Mississippi State Mississippi USA

**Keywords:** estuary, forage finfish, Gulf of Mexico, land‐use change, pelagic fish, shrimp, species richness

## Abstract

The composition of land use/land cover (LULC) in coastal watersheds has many implications for estuarine system ecological function. Land use/land cover can influence allochthonous inputs and can enhance or degrade the physical characteristics of estuaries, which in turn affects estuaries' ability to support local biota. However, these implications for estuaries are often poorly considered when assessing the value of lands for conservation. The focus of research regarding terrestrial and estuarine interfaces often evaluates how LULC may stress estuarine ecosystems, but in this study we sought to understand how LULC may both positively and negatively affect estuaries using measures of observed biotic richness as proxies for estuarine function. We investigated the influence of LULC on estuarine biotic richness with Bayesian hierarchical models using multiple geospatial data sets from 33 estuaries and their associated watersheds along the Gulf of Mexico coastal region of the United States. We designed the hierarchical models with observed species richness of three functional groups (FGs) (i.e., pelagic fishes, forage fishes, and shrimp) from fishery‐independent trawl surveys as response variables. We then set salinity and water temperature as trawl‐specific covariates and measures of influence from six LULC classes as estuary‐specific covariates and allowed the models to vary by estuary, trawl program, salinity, and temperature. The model results indicated that the observed richness of each FG was both positively and negatively associated with different LULC classes, with estuarine wetlands and forested lands demonstrating the strongest positive influences on each FG. The results are generally consistent with past studies, and the modeling framework provides a promising way to systematically quantify LULC linkages with the biotic health of estuaries for the purposes of potentially valuing the estuarine implications of land conservation.

## INTRODUCTION

Land use/land cover (LULC) influences many aspects of ecological function within estuarine systems, which subsequently influences an estuary's capacity to support local biota. Natural and anthropogenic LULC often have conflicting impacts on estuaries, where natural LULC is defined as any land that is not developed or used for agricultural purposes, with the exception of land used for silviculture. Natural LULC regulates nutrient, sediment, and coarse particulate organic matter (CPOM) inputs into estuaries (Basnyat et al., 1999; Carter, 1996; Engle, 2011; Morgan et al., 2009) and can also provide structural protection to estuaries from wind and wave energy associated with episodic weather events or persistent wave action (Costanza et al., 2008; USACOE, 1963; Wamsley et al., 2010). Conversely, anthropogenic LULC can supersede, modify, or eliminate some of these regulatory provisions and result in deleterious responses in estuaries (Cox & Preda, 2005; Kemp et al., 1983; Stoms et al., 2005; Thrush et al., 2004). For example, Short and Burdick (1996) demonstrated eel grass loss as a function of housing development and nutrient loading in Waquoit Bay, Massachusetts. Diverted sediment loading in the Mississippi River Delta from levee and canal construction was also shown to accelerate salt marsh degradation and conversion to open water (Turner, 1990). An assessment of fertilized land uses within estuary catchments in Australia also found shifts in the proportion of seagrass to macroalgae presence within estuaries (Cook et al., 2018).

In addition to affecting overall estuarine function, changes in LULC may also impact the structure and function of biotic communities using estuarine resources for all or part of their life history. Some changes result in direct population losses, while others elicit shifts in community composition. For example, the King George whiting (*Sillaginodes punctatus*) fishery in North Spencer Gulf, Australia, sustained a 40% loss in commercial landings following a 16% reduction in seagrass habitat caused by anthropogenically linked increases in turbidity that decreased light penetration (McArthur & Boland, 2006). A study on how the anthropogenic influence of groundwater affects coral reef communities in the Hawaiian archipelago revealed that increases in nutrient loading had the potential to negatively impact browsers and piscivores within the reef fish community, and groundwater discharge influenced reef structure (Delevaux et al., 2018).

Despite all that is known about linkages between terrestrial and aquatic systems, the implications of surrounding LULC on estuarine health are often ineffectively considered in valuing opportunities for land conservation (Álvarez‐Romero et al., 2011; Beck, 2003; Klein et al., 2012). Given the ecosystem services that coastal and near‐coastal lands provide for estuarine health, the effects of land composition on an estuary's freshwater inputs, structural resilience, and habitat provisions must also have a measurable association with biotic health within an estuary. Despite the extensive documentation of ecological processes that relate land use to estuaries (Bilkovic et al., 2006; Miller et al., 2018; Short & Burdick, 1996; Thrush et al., 2004), limited effort has been devoted to defining the value of LULC as it relates to estuarine biota (Álvarez‐Romero et al., 2015; Delevaux et al., 2018). Such efforts could provide a means to value potential land conservation actions based on how an area of interest associates with a component of estuarine biotic health.

Several approaches to characterizing associations of LULC with biotic health have been proposed, including measures of species richness, diversity indices, indices of biotic integrity, and phylogenetic diversity (Brown et al., 2019; Graham et al., 2019; Miller et al., 2018; Wang et al., 1997). Modeling the linkage between LULC and estuarine biotic health requires two major considerations: structuring the model and characterizing the response variable. Processes to incorporate in modeling land–sea relationships may include hydrologic (Álvarez‐Romero et al., 2015), biogeochemical (Delevaux et al., 2018), and pollutant dispersal parameters (Rude et al., 2016), all of which have complexities that need to be considered before determining the appropriateness for inclusion. The availability of data to adequately describe these ecological processes is one such factor for consideration. Estuaries and their associated catchments contain ecological processes that are hierarchical in nature. Thus, models within these systems should be structured to adequately reflect these natural hierarchies. However, choosing appropriate metrics for a response variable is a recurrent methodological challenge. Traditional taxonomic diversity metrics, it is claimed, are insufficient indicators of a conserved area's value in supporting ecological function (Jarzyna & Jetz, 2016). However, the species richness of functional groups (FGs), rather than taxonomic groups, may be a valuable metric in understanding the effects of LULC on biotic composition and, ultimately, ecological function within estuaries. FGs, as defined here, categorize species based on broadly defined niches that may imply similar sensitivities to environmental characteristics.

Fisheries are a fundamental component of the economy and culture within the Gulf of Mexico Coastal Region (GCR) of the United States and are thus worth protecting through direct and indirect actions. An estimated 2.7 million recreational anglers went fishing in 2016 in the GCR (National Marine Fisheries Service, 2018), and almost 20% of the seafood caught in the United States comes from fisheries in the Gulf of Mexico (National Marine Fisheries Service, 2018). Because of the importance of commercial and recreational fishing to this region, much attention is given to the management of fishery resources by state and federal agencies. However, opportunities to extend protections to fisheries in the GCR through less direct means, such as land acquisition or easement, may be feasible given the known benefits that natural LULC may provide estuaries (Álvarez‐Romero et al., 2011; Carter, 1996; Engle, 2011). Given the value that the best available science may provide to improving the efficacy of conservation and restoration, we thus seek to quantify the value of land conservation along the GCR as it pertains to the health of estuarine biotic communities.

Protecting offsite ecosystem services is an intended function of land conservation, and protecting estuarine resources is a major goal of the Resources and Ecosystems Sustainability, Tourist Opportunities, and Revived Economies (RESTORE) Council and its member agencies. Despite abundant research on the effects of LULC on the environmental quality of estuarine ecosystems (Bilkovic et al., 2006; Dauer et al., 2000; Delpech et al., 2010; Lowe & Peterson, 2014; Miller et al., 2018; Short & Burdick, 1996), much of the focus has centered on characteristics of watersheds that function as system stressors (e.g., Miller et al., 2018). While it is assumed that natural landscapes provide ecosystem services to estuaries, there is no systematic approach to quantifying associations of estuarine health with the surrounding LULC. Thus, our goal was to quantify the influence of coastal and near‐coastal LULC on the biotic richness of estuaries, such that predictions regarding the effects of land conservation actions on estuarine biotic communities could be ultimately derived. Our objective in this study was to predict the impact of coastal and near‐coastal watershed landscapes on the expected species richness of aquatic organisms within estuaries by developing appropriately parameterized hierarchical models, using the GCR complex of estuaries as an example. We postulated that the influence of land cover on estuarine aquatic ecosystems was associated with the amount of surface water runoff in contact with each land‐cover type in a given watershed. We suggest that the greater amount of runoff that originates from a particular LULC class, the more water with that LULC class's imprint will end up downstream and, thus, have a greater effect on the estuary's water quality and profile and aquatic biota.

## METHODS

**FIGURE 1:**
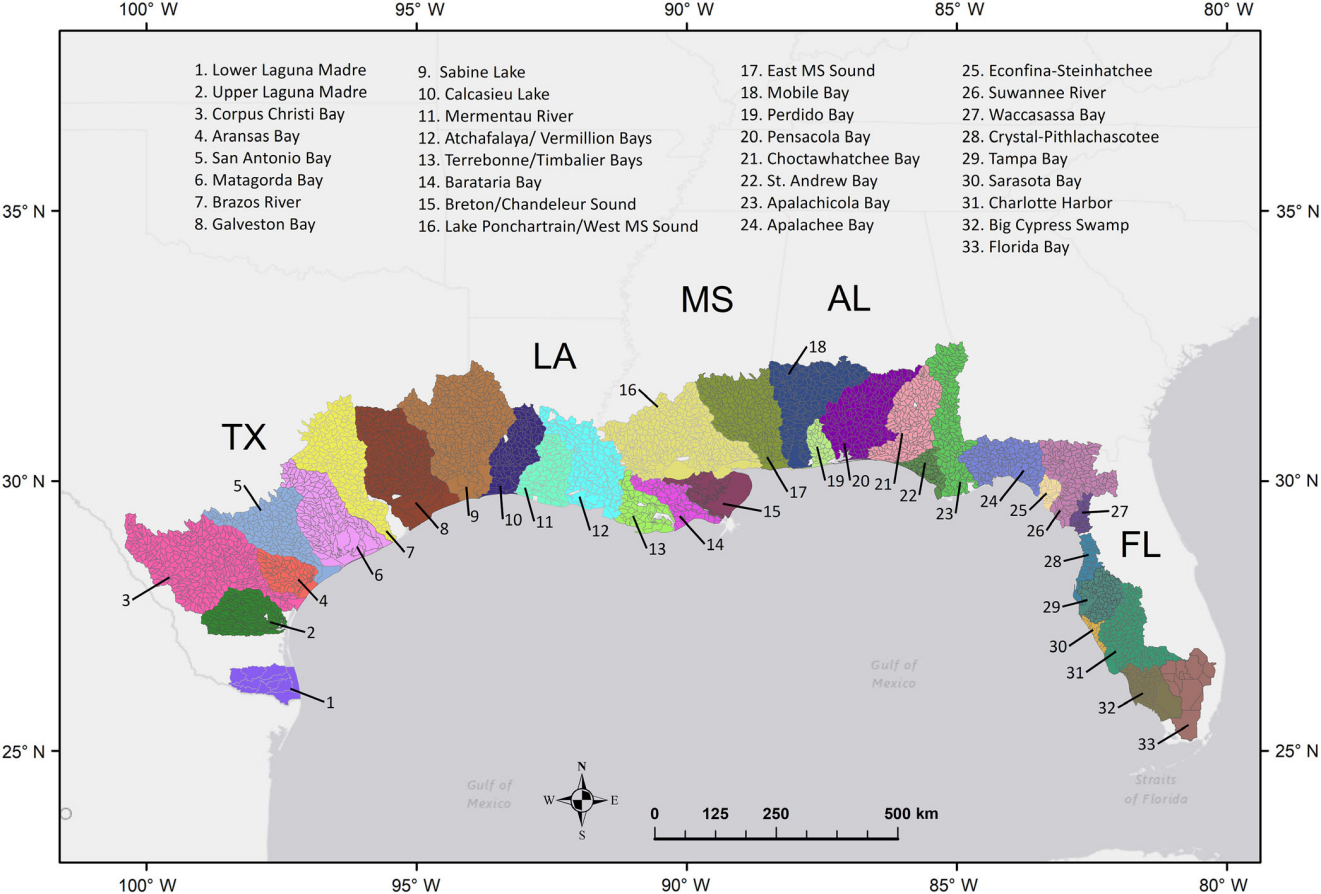
Catchment areas used for the 33 estuaries included in the study. The estuaries are within the five states along the Gulf Coast Region (GCR) of the United States: Texas (TX), Louisiana (LA), Mississippi (MS), Alabama (AL), and Florida (FL). All HUC12 subbasins included in the study are delineated and are the watershed areas used to calculate mean annual runoff volume contributed by each land use/land cover (LULC) class. Basemap courtesy of OpenStreetMap.

We constructed three hierarchical models across 33 GCR estuaries and their associated watersheds (Figure 1) that estimate the expected number of species observed in a trawl sample based on temperature, salinity, and runoff volume per catchment area of six different LULC classes. We delineated estuaries using existing features of estuarine drainage area, coastal drainage area, and fluvial drainage area data from the Coastal Assessment Framework by the National Oceanic and Atmospheric Administration (NOAA) (Borchert et al., 2018). We used data from trawl samples (*n* = 68,921) collected from 1991 through 2009 by seven trawl programs, including one program from each of the five state agencies (Florida, Alabama, Mississippi, Louisiana, and Texas), the U.S. Environmental Protection Agency (EPA) Environmental Monitoring and Assessment Program (EMAP), and the National Coastal Assessment (NCA) program (Appendix S1: Table S1). Trawl sample data included counts of individual species, mean salinity (ppt), and water temperature (°C). We excluded trawl samples that appeared to have outliers, including removing sampling events with temperatures below 5°C or above 35°C and any negative salinity values. We then categorized subsets of species into three FGs (pelagic, forage finfish, shrimp; Table 1), based on broadly defined niches such as community function and life histories that may imply similar sensitivities to environmental characteristics (Petchey & Gaston, 2006). For instance, the pelagic FG primarily contained species in the Scombridae family and species found in the order Carangiformes, as these species are often piscivorous and found in open water but are also often found in estuaries seasonally or sometimes in their juvenile stages.

**TABLE 1:** List of taxa, and their functional group, observed within the final subset of trawl samples used in the Bayesian hierarchical models.

Scientific name	Common name	Functional group	No. of trawls observed
*Clupeidae*	Herring	Forage finfish	1
*Alosa alabamae*	Alabama shad	Forage finfish	1
*Alosa chrysochloris*	Skipjack herring	Forage finfish	3
*Clupea harengus*	Atlantic herring	Forage finfish	1
*Brevoortia*	Menhadens	Forage finfish	1
*Brevoortia gunteri*	Finescale menhaden	Forage finfish	2
*Brevoortia patronus*	Gulf menhaden	Forage finfish	744
*Dorosoma cepedianum*	Gizzard shad	Forage finfish	51
*Dorosoma petenense*	Threadfin shad	Forage finfish	260
*Opisthonema oglinum*	Atlantic thread herring	Forage finfish	31
*Harengula jaguana*	Scaled sardine	Forage finfish	62
*Sardinella aurita*	Spanish sardine	Forage finfish	1
*Anchoa*	Common anchovies	Forage finfish	8
*Anchoa hepsetus*	Striped anchovy	Forage finfish	207
*Anchoa mitchilli*	Bay anchovy	Forage finfish	1941
*Anchoa lyolepis*	Dusky anchovy	Forage finfish	20
*Anchoa nasuta*	Longnose anchovy	Forage finfish	11
*Membras martinica*	Rough silverside	Forage finfish	2
*Menidia*	Menidia silversides	Forage finfish	6
*Menidia beryllina*	Inland silverside	Forage finfish	7
*Menidia peninsulae*	Tidewater silverside	Forage finfish	3
*Mugil cephalus*	Striped mullet	Forage finfish	141
*Polydactylus octonemus*	Atlantic threadfin	Forage finfish	62
*Peprilus paru*	Harvestfish	Forage finfish	92
*Peprilus triacanthus*	Butterfish	Forage finfish	1
*Peprilus burti*	Gulf butterfish	Forage finfish	190
*Engraulidae*	Anchovies	Forage finfish	1
*Pomatomus saltatrix*	Bluefish	Pelagic	1
*Rachycentron canadum*	Cobia	Pelagic	2
*Caranx hippos*	Crevalle jack	Pelagic	78
*Caranx latus*	Horse‐eye jack	Pelagic	2
*Caranx crysos*	Blue runner	Pelagic	2
*Chloroscombrus chrysurus*	Atlantic bumper	Pelagic	294
*Oligoplites saurus*	Leatherjack	Pelagic	7
*Selene vomer*	Lookdown	Pelagic	103
*Selene setapinnis*	Atlantic moonfish	Pelagic	72
*Trachinotus carolinus*	Florida pompano	Pelagic	8
*Trachinotus falcatus*	Permit	Pelagic	1
*Decapterus punctatus*	Round scad	Pelagic	1
*Hemicaranx amblyrhynchus*	Bluntnose jack	Pelagic	22
*Trichiurus lepturus*	Atlantic cutlassfish	Pelagic	197
*Scomberomorus cavalla*	King mackerel	Pelagic	1
*Scomberomorus maculatus*	Spanish mackerel	Pelagic	41
*Sphyraena guachancho*	Guaguanche	Pelagic	8
*Sphyraena borealis*	Northern sennet	Pelagic	2
*Echeneis naucrates*	Sharksucker	Pelagic	3
*Echeneis neucratoides*	Whitefin sharksucker	Pelagic	1
*Elops saurus*	Ladyfish	Pelagic	35
*Megalops atlanticus*	Tarpon	Pelagic	1
*Centropomus undecimalis*	Common snook	Pelagic	1
*Penaeidae*	Penaeid shrimp	Shrimp	1
*Xiphopenaeus kroyeri*	Seabob	Shrimp	63
*Acetes americanus*	Sergestid shrimp	Shrimp	9
*Sicyonia brevirostris*	Brown rock shrimp	Shrimp	6
*Sicyonia dorsalis*	Lesser rock shrimp	Shrimp	10
*Sicyonia laevigata*	Hardback	Shrimp	1
*Macrobrachium*	River shrimp	Shrimp	1
*Macrobrachium ohione*	River or Ohio shrimp	Shrimp	62
*Palaemonetes*	Grass shrimp spp.	Shrimp	147
*Palaemonetes intermedius*	Grass shrimp	Shrimp	1
*Palaemonetes paludosus*	Eastern grass shrimp	Shrimp	3
*Palaemonetes pugio*	Daggerblade grass shrimp	Shrimp	23
*Palaemonetes vulgaris*	Caridean grass shrimp	Shrimp	144
*Palaemon floridanus*	Florida grass shrimp	Shrimp	1
*Alpheidae*	Snapping shrimp	Shrimp	1
*Alpheus heterochaelis*	Bigclaw snapping or pistol shrimp	Shrimp	5
*Alpheus estuariensis*	Estuarine snapping shrimp	Shrimp	11
*Hippolyte zostericola*	Zostera shrimp	Shrimp	1
*Lysmata wurdemanni*	Peppermint shrimp	Shrimp	3
*Tozeuma carolinense*	Arrow shrimp	Shrimp	16
*Rimapenaeus*	Roughneck shrimp	Shrimp	8
*Farfantepenaeus aztecus*	Brown shrimp	Shrimp	1255
*Farfantepenaeus duorarum*	Pink shrimp	Shrimp	578
*Rimapenaeus constrictus*	Roughneck shrimp	Shrimp	30
*Rimapenaeus similis*	Roughback shrimp	Shrimp	52
*Litopenaeus setiferus*	White shrimp	Shrimp	1277
*Farfantepenaeus*	Commercial shrimp	Shrimp	63

We defined a LULC class's influence within each estuary's watershed as the mean annual volume of runoff based on 30 years of precipitation data (1981–2010), originating from the LULC class, normalized by the area of each watershed analyzed. To calculate annual runoff volume, we used the curve number method described by the Natural Resources Conservation Service (NRCS) Technical Report 55 (SCS, 1986), which required data for LULC, soil hydrologic group, and precipitation. We acquired LULC data for each estuary's watershed from the NOAA Coastal Change Analysis Program (C‐CAP) 2001 Regional Land Cover (Office for Coastal Management, 2019) with a resolution of 30 × 30 m. We chose the 2001 C‐CAP layer because it represents the midrange of the trawl sampling period (1991–2009). Despite the inherent changes in land cover over the course of trawl sample collections, Homer et al. (2007) found that the 1992, 2001, and 2006 versions of the National Land Cover Database were highly correlated (*r* > 0.98), indicating that the land composition between the beginning and end of the study was likely relatively similar in the C‐CAP mapping effort. With the exception of barren and cultivated cropland, LULC class coefficients were aggregates of multiple C‐CAP 2001 land‐cover classes (Table 2). We obtained the soil hydrologic group from the Soil Survey Geographic Database (SSURGO), which houses soil data collected over the last century by the National Cooperative Soil Survey (USDA/NRCS, 2019). Lastly, we acquired precipitation data from the Parameter‐Elevation Regressions on Independent Slopes Model (PRISM) Climate Group (Di Luzio et al., 2008), which specified daily precipitation for the study region (Figure 1) from 1981 to 2010. Using combinations of LULC class and soil hydrologic group, we generated curve number values for the landscape found in the technical guide of NOAA's Nonpoint Source Pollution and Erosion Comparison Tool (OpenNSPECT) (NOAA, 2014) for all LULC classes except wetlands. We opted to replace the curve number values for all palustrine and estuarine wetland classes from 0, indicating no runoff, to 100, indicating no infiltration, since there would be no way to compare wetland influence across estuaries if they were assumed to generate no runoff.

**TABLE 2:** Outline of parameters used in models and summary of Coastal Change Analysis Program (C‐CAP) land‐cover attributes aggregated to create corresponding parameters.

Parameter	Name	C‐CAP attribute (if applicable)
X1	Mean salinity	Not applicable
X2	Mean temperature	Not applicable
X3	Developed	2 High intensity developed
		3 Medium intensity developed
		4 Low intensity developed
		5 Developed open space
X4	Cropland	6 Cultivated land
X5	Forest	9 Deciduous forest
		10 Evergreen forest
		11 Mixed forest
X6	Palustrine wetland	13 Palustrine forested wetland
		14 Palustrine scrub/shrub wetland
		15 Palustrine emergent wetland
X7	Estuarine wetland	16 Estuarine forested wetland
		17 Estuarine scrub/shrub wetland
		18 Estuarine emergent wetland
X8	Barren	20 Bare land

We developed a series of Bayesian hierarchical models for each FG to quantify associations of species richness with surrounding land cover characteristics. We used species count by FG as response variables and salinity (ppt) and water temperature (°C) measured during each trawl sample as site‐level fixed effects. We included runoff volume per LULC class (developed, barren, palustrine wetlands, estuarine wetlands, cropland, forest) within each watershed, normalized by watershed area (m^3^/km^2^), as group‐level fixed effects. We also included estuary ID and trawl‐sampling program as random intercepts in each model to acknowledge unmeasured variance associated with samples in those groups and included salinity and temperature as random slopes because they exhibited distinct distributions across estuaries and trawl programs. We structured the overall model for each FG as a multilevel Poisson‐lognormal model described as follows by Equation (1)

(1) $$\begin{array}{ll} \log\left(Y_{j,k}\right) & =\beta_{0}+\sum_{i=1}^{8}\left(\beta_{i}∙X_{i}\right)+\alpha_{0,j}+\sum_{i=1}^{2}\left(\alpha_{i,j}∙X_{i}\right)\\ & +\;\alpha_{0,k}+\sum_{i=1}^{2}\left(\alpha_{i,k}∙X_{i}\right), \end{array}$$

where $Y_{j,k}$ is the expected count of species in a FG from estuary *j* and trawl program *k*, $\beta_{1}$ and $\beta_{2}$ are the coefficients for sample‐level mean salinity and water temperature, and $\beta_{3}$ through $\beta_{8}$ are estuary‐level coefficients for the effects of LULC classes (developed, cultivated cropland, palustrine wetland, estuarine wetland, barren, and forest). We log‐transformed and group‐mean‐centered all sample‐level covariates and log‐transformed and grand‐mean‐centered all estuary‐level covariates in each model. The estuary (*j*) and trawl program (*k*) were expressed as random intercepts $\alpha_{0,j}$ and $\alpha_{0,k}$, respectively, and salinity and temperature were also included as random slopes by estuary and trawl program, seen as $\alpha_{1,j}$, $\alpha_{2,j}$, $\alpha_{1,k}$, and $\alpha_{2,k}$, respectively.

To implement a Bayesian model, we specified model diagrams in JAGS code (Plummer, 2017), including joint and posterior distributions (Equation 2), as follows:

(2) $$\left[\lambda ,\sigma_{0}^{2},\sigma_{s}^{2},\beta ,\rho ,\alpha ,\sum |Y\right]\propto \prod_{j=1}^{33}\prod_{k=1}^{7}\text{Poisson}\left[Y_{j,k}|\lambda_{j,k},\sigma_{0}^{2}\right]•\text{lognormal}\left(\lambda_{j,k},|,\frac{g{\left(\alpha_{j},\alpha_{k},\beta ,x_{j,k}\right)}^{2}}{\sigma_{s,j,k}^{2}},\frac{g\left(\left(\alpha_{j},\alpha_{k},\beta ,x_{j,k}\right)\right)}{\sigma_{s,j,k}^{2}}\right)•\text{inverse gamma}\left(\sigma_{s,j,k}^{2}|.001,.001\right)•\text{multivariate normal}(\alpha_{j}\left|\rho ,\sum_{1}\right)•\text{multivariate normal}(\alpha_{k}\left|\rho ,\sum_{2}\right)•\text{multivariate normal}\left(\beta |\rho ,\sum_{3}\right)•\prod_{q=0}^{2}\text{inverse Wishart}\left(\sum_{q}|\Omega ,p\right)•\prod_{h=0}^{2}\text{normal}\left(\rho_{h}|0,\tau \right)•\prod_{h=0}^{2}\text{half}-\text{Cauchy}\left(\tau_{h}|0,4^{2}\right)•\prod_{v=0}^{2}\text{normal}\left(\rho_{v}|0,\tau \right)•\prod_{v=0}^{2}\text{half}-\text{Cauchy}\left(\tau_{v}|0,4^{2}\right)•\prod_{m=0}^{8}\text{normal}\left(\rho_{m}|0,\tau \right)•\prod_{m=0}^{8}\text{half}-\text{Cauchy}\left(\tau_{m}|0,4^{2}\right),$$

where the expected species count $\lambda_{j,k}$ from estuary *j* and trawl program *k* was modeled as a lognormal distribution of fixed effects β and slopes and intercepts that varied by estuary and trawl program. We modeled the prior distributions of the fixed effects as $\text{multivariate normal}\left(\beta |\rho ,\sum_{3}\right)$, where ρ was a vector of means and $\sum_{3}$ was a variance–covariance matrix, since we could not assume that they were independent. We also assigned random slopes and intercepts multivariate normal prior distributions. We selected normal distributions for the means **ρ**_*m*_, **ρ**_*h*_, and **ρ**_*v*_ of the fixed, estuary, and trawl program effect priors, respectively. We also assigned half‐Cauchy hyperpriors to the variances $\tau_{m}$, $\tau_{h}$, and $\tau_{v}$. We provided weakly‐informative priors to all parameters within the model (Appendix S1).

We implemented the model structure described in Equations (1) and (2) into three Markov chain Monte Carlo (MCMC) simulations to infer species counts of the three FGs to landscape characteristics and trawl‐specific controls. We constructed the MCMC algorithms within JAGS version 4.3.0 (Plummer, 2017) and ran them with the autorun.jags() function in R version 3.6.0 statistical software (R Core Team, 2019). We used a subset of the 68,921‐sample data set to improve the run time of the MCMC simulation models. Subsets were taken from each estuary that had more than 150 trawl events, where 150 trawls were sampled without replacement and graphically checked to assure the subsets had probability distributions that were similar to those of the original data set. We determined the convergence of the models with the Gelman‐Rubin statistic using a threshold value of <1.10 for each parameter, and sample lengths were calculated based on Raftery and Lewis's diagnostic. Upon completion of the MCMC algorithms, we used the posterior distributions of each landscape covariate to quantify associations between the expected influence of each land‐cover type and species richness within trawl samples for each FG.

To assess model fit, we implemented posterior predictive checks to investigate whether we had evidence that our models did not properly represent the data (Conn et al., 2018). To calculate the posterior predictive check, we used the following integral (Equation 3):

(3) $$P_{B}=\mathrm{Pr}\left(T\left(y^{\mathrm{new}},\theta \right)\geq T\left(y,\theta \right)\right)=\iint I_{\left\{T\left(y^{\mathrm{new}},\theta \right)\geq T\left(y,\theta \right)\right\}}\left[y^{\mathrm{new}}|\theta \right]\left[\theta |y\right]dy^{\mathrm{new}}\mathrm{d\theta},$$

where $P_{B}$ is known as the Bayesian *p* value, which assesses whether the distribution of data predicted ($y^{\mathrm{new}}$) by the model is similar or not to the observed data. A Bayesian *p* value between 0.1 and 0.9 indicated no evidence for a model's lack of fit. Model validations were also conducted by calculating a Bayesian *p* value to assess the similarity of the fitted model predictions against the 65,302 trawl samples not used in model fitting. All R and JAGS code used to run the Bayesian models and model checking procedures is available in Data S1 with descriptions in Metadata S1.

## RESULTS

**FIGURE 2:**
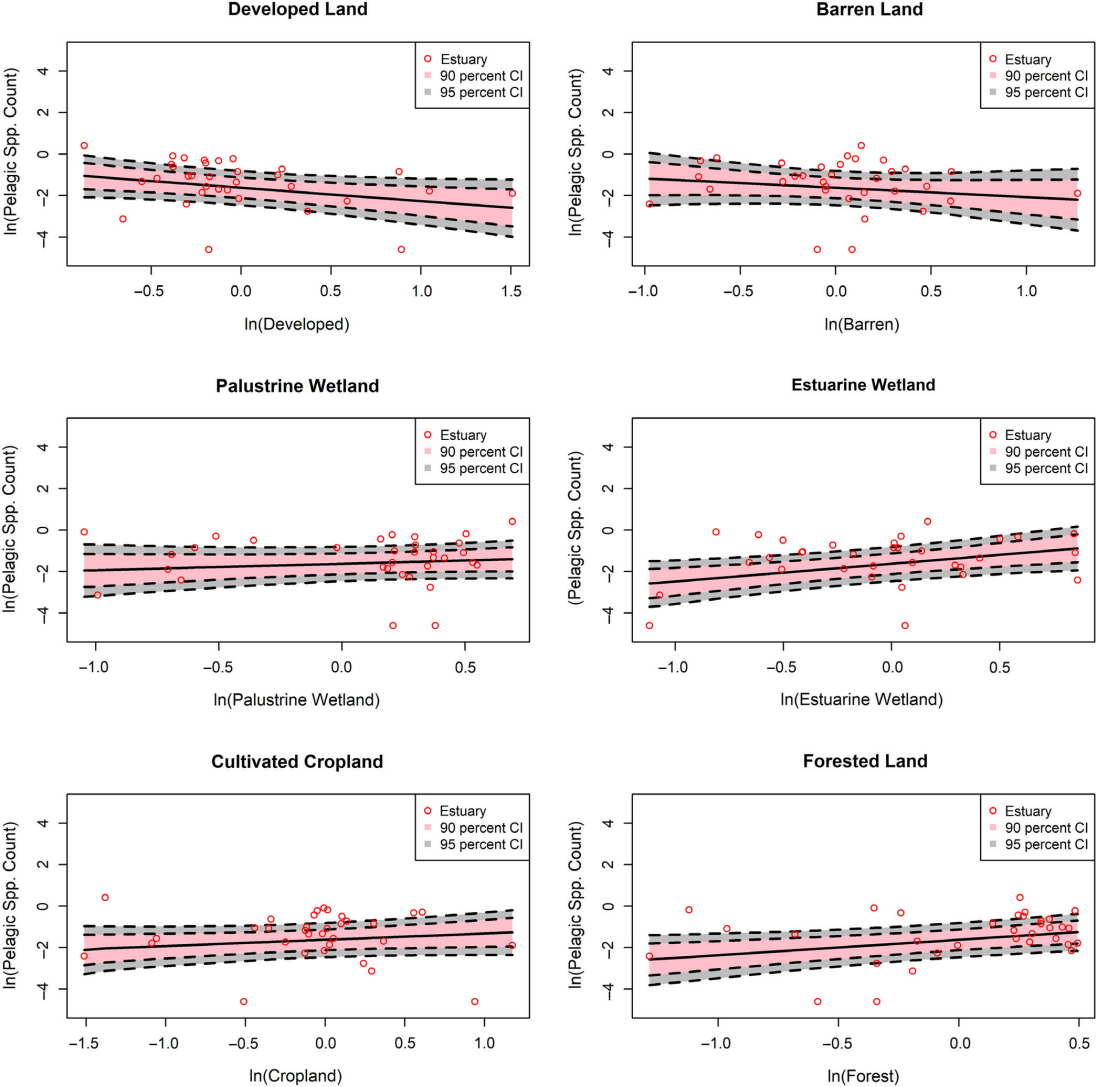
Log‐normalized associations of expected pelagic species richness from trawl samples with change in influence from each land use/land cover (LULC) group. Each graph shows mean pelagic species richness observed in each estuary, mean expected richness, and 90% and 95% credible intervals of expected richness. LULC group data were log‐transformed and grand‐mean centered.

**FIGURE 3:**
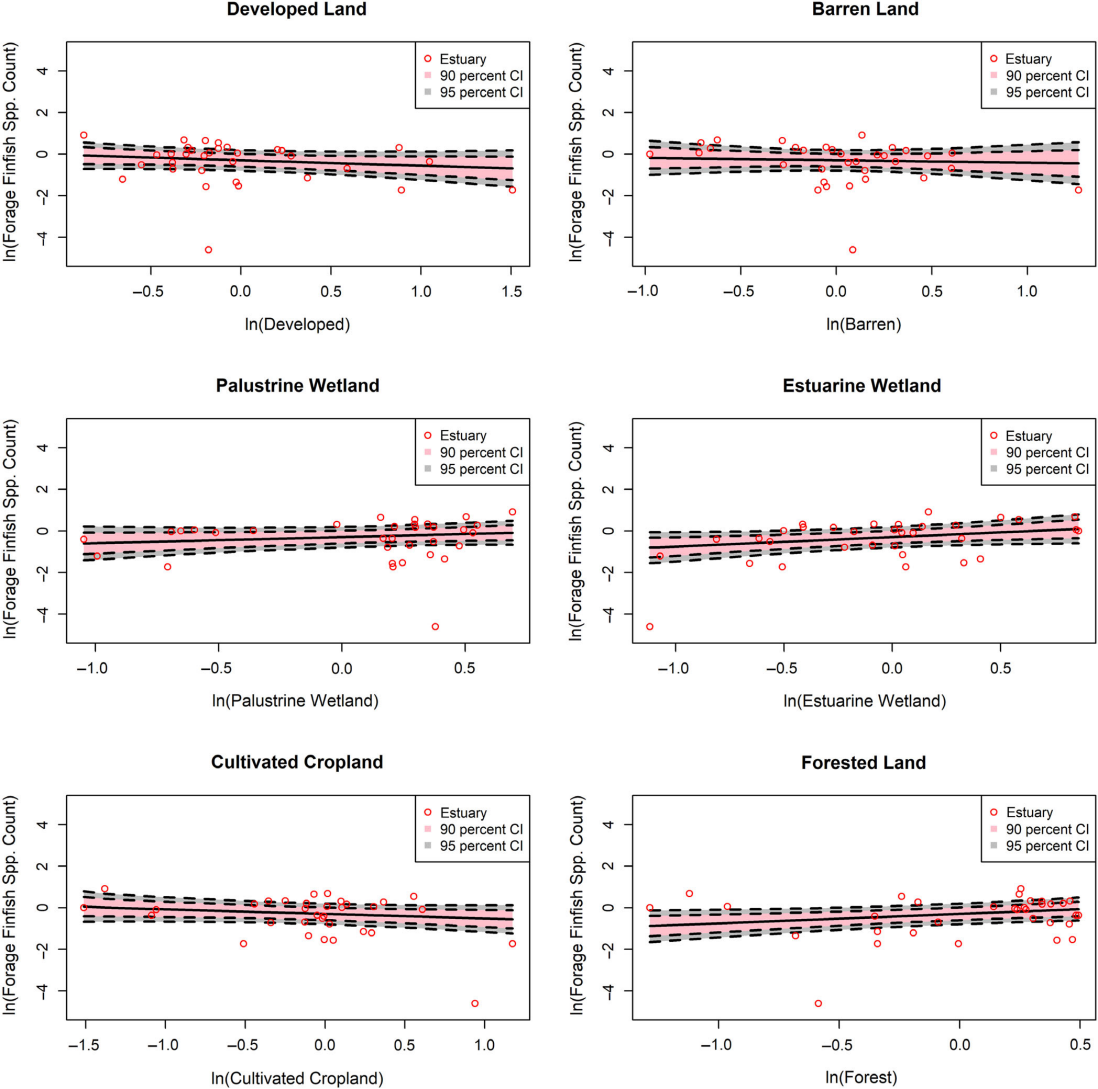
Log‐normalized associations of expected forage finfish species richness from trawl samples with change in influence from each land use/land cover (LULC) group. Each graph shows mean forage finfish species richness observed in each estuary, mean expected richness, and 90% and 95% credible intervals of expected richness. LULC group data were log‐transformed and grand‐mean centered.

**FIGURE 4:**
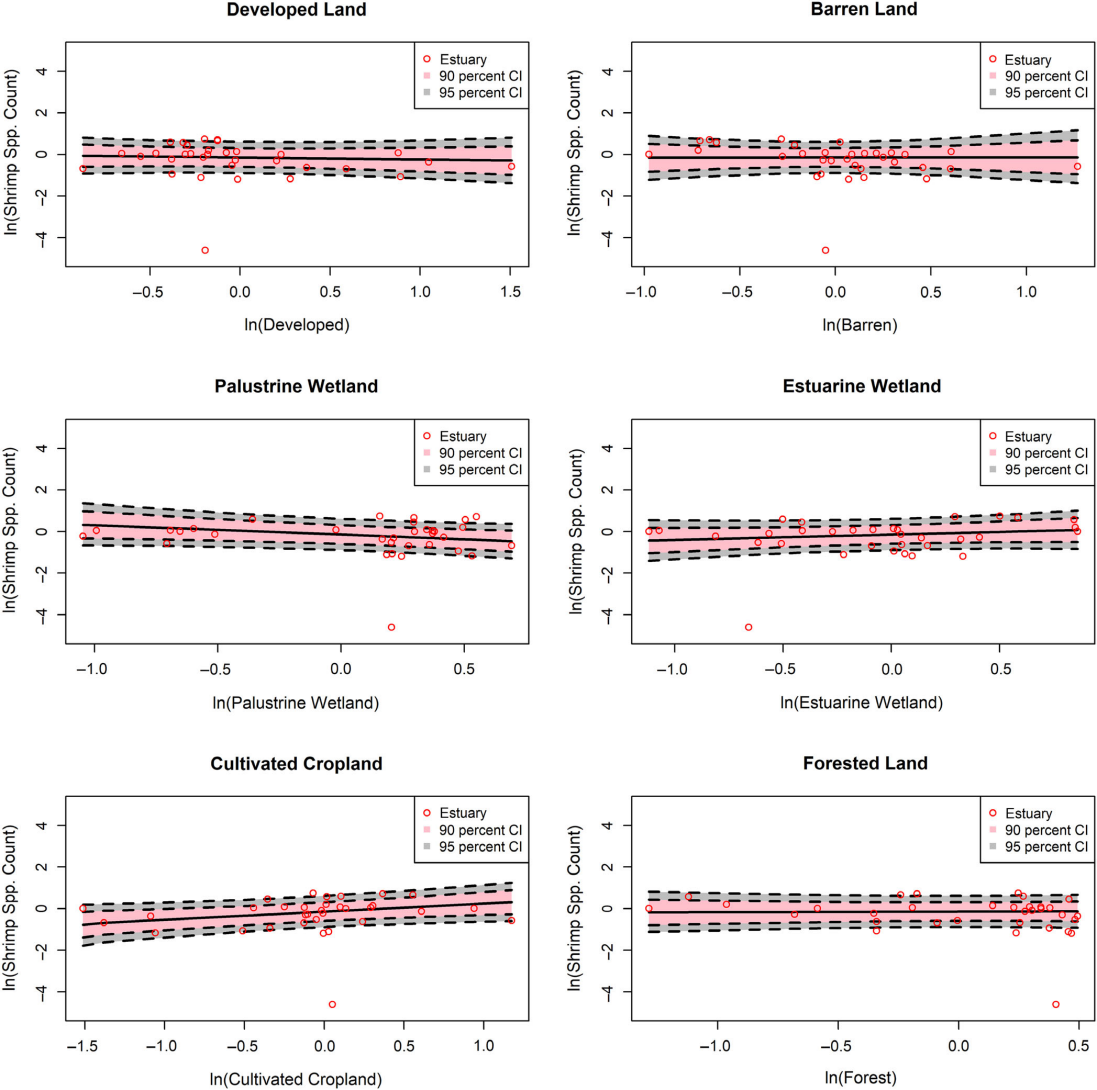
Log‐normalized associations of expected shrimp species richness from trawl samples with change in influence from each land use/land cover (LULC) group. Each graph shows mean shrimp species richness observed in each estuary, mean expected richness, and 90% and 95% credible intervals of expected richness. LULC group data were log‐transformed and grand‐mean centered.

Within the subsetted data, a combined total of 84 species (23 pelagic, 23 forage finfish, and 27 shrimp) were observed in our subset of 3619 trawl samples (Table 1). Species counts within trawl samples generally exhibited positive associations with estuaries that had greater influence from natural LULC classes (i.e., palustrine wetland, estuarine wetland, forest) and negative associations with estuaries that had greater influence from developed LULC classes (Figures 2, 3, 4 and Table 3). Associations with cultivated cropland depended on FG, with shrimp richness showing positive associations and forage finfish showing weakly negative associations with cultivated cropland cover. Species counts of all FGs were most positively associated with estuarine wetland classes. Pelagic and forage finfish species counts were also positively associated with forest classes, whereas shrimp appeared to lack an association with forest land cover. Shrimp species richness in trawl samples showed weak associations with most land‐cover class groups and only positively associated with increasing cultivated cropland influence and, to a lesser degree, estuarine wetland.

**TABLE 3:** Summary of 2.5th, 50th, and 97.5th percentile coefficients of each Bayesian credible interval by functional group and LULC covariate.

Functional group	Covariate	Percentile		
		2.50th	50th	97.50th
Pelagic	Developed	−1.277	−0.487	0.295
	Barren	−1.510	−0.415	0.672
	Palustrine wetland	−0.619	0.299	1.193
	Estuarine wetland	0.022	0.860	1.692
	Cultivated cropland	−0.165	0.454	1.071
	Forest	0.049	0.790	1.601
Forage Finfish	Developed	−0.747	−0.264	0.207
	Barren	−0.824	−0.118	0.577
	Palustrine wetland	−0.259	0.309	0.846
	Estuarine wetland	−0.075	0.451	1.005
	Cultivated cropland	−0.582	−0.220	0.151
	Forest	−0.027	0.453	0.954
Shrimp	Developed	−0.608	−0.073	0.445
	Barren	−0.811	−0.024	0.812
	Palustrine wetland	−1.108	−0.452	0.170
	Estuarine wetland	−0.337	0.243	0.856
	Cultivated cropland	−0.035	0.378	0.822
	Forest	−0.461	0.046	0.530

In general, the pelagic FG exhibited the greatest magnitudes of association with each land‐cover group. For instance, between the lowest and highest developed land influence observed across estuaries, expected pelagic species richness declined by 73.2%, while forage finfish and shrimp species richness declined by 43.9% and 15.5%, respectively (Table 4). Likewise, between the lowest and highest estuarine wetland influence, expected pelagic species richness increased by 453%, and expected richness of forage finfish and shrimp increased by 143% and 64%, respectively (Table 4).

**TABLE 4:** Proportional change in expected species richness of each functional group as land cover changes from lowest to highest amount of influence observed across estuaries.

Covariate	Functional group		
	Pelagic	Forage finfish	Shrimp
Developed	−0.732	−0.439	−0.155
Barren	−0.523	−0.189	0.081
Palustrine wetland	0.437	0.610	−0.570
Estuarine wetland	4.539	1.438	0.644
Cultivated cropland	2.721	−0.458	1.980
Forest	2.743	1.207	0.006

The variances of random intercepts and slopes and effect sizes of fixed effects were reported for each FG (Appendix S1: Table S5 and S6, respectively). Random intercept variances indicate that estuary and trawl program have a moderate to large effect on explaining species richness, especially for pelagic species richness. For the pelagic group, increases in salinity and temperature have a moderately positive effect on explaining species richness. Correlation tables were generated for each model (Appendix S1: Tables S2–S4), which indicate some correlations between LULC parameter estimates, with barren and developed classes showing the strongest correlation for all three models (<−0.60). With the exception of barren and developed classes, correlations between covariates were between −0.5 and 0.5, which should not appreciably hinder the interpretation of model results.

For the three models, convergence was determined both through visual inspection of trace plots and with Gelman and Rubin diagnostics with threshold values of <1.10 for each parameter. Raftery and Lewis diagnostics determined the number of iterations to run each model to estimate the 2.5% quantile with an accuracy of 0.005 and a 95% probability. Posterior predictive checks for each model resulted in Bayes *p* values of 0.526, 0.492, and 0.502 for the pelagic, forage finfish, and shrimp model, respectively. Because these values are well within 0.1 and 0.9, the posterior predictive checks indicate no evidence for lack of fit of the model. The Bayes *p* values from the additional 65,302 samples were 0.824 for the pelagic model, 0.608 for the forage finfish model, and 0.461 for the shrimp model, indicating sufficient predictive ability. The codebase of each model and the posterior predictive checks are available in Data S1 and Metadata S1.

## DISCUSSION

The patterns observed in the models are generally consistent with the ecosystem services and disturbances associated with land covers represented in the model (Boesch & Turner, 1984; Engle, 2011; Goetz et al., 2004). Though this research supports previous findings, the novelty in our approach is that it elicits empirical affirmations of associations between LULC and estuarine biotic health, which were previously based on anecdotal information (Beger et al., 2010; Stoms et al., 2005; Thrush et al., 2004). Whereas previous studies quantified effects of anthropogenic stressors on estuarine health/condition (e.g., Miller et al., 2018; Teichert et al., 2016), our study also quantified the linkage between natural land covers and estuarine biotic health. Establishing a quantifiable link between all land‐cover types (anthropogenic and natural) and estuarine health is a critical step in systematically valuing implications of land conservation to estuarine environments. Our approach only considered how the type of LULC relates to aquatic species richness, whereas other studies considered more nuanced aspects of the landscape such as nutrient loads and groundwater movement (Delevaux et al., 2018). While more detailed information about the landscape may better explain processes from the land that relate to the marine environment, the information needed for these approaches is more difficult to obtain than our approach, and therefore such approaches may be harder to replicate for other regions.

Here we used species richness within FGs of aquatic organisms as bioindicators of estuarine health because these primary, secondary, and sometimes tertiary consumers may have similar environmental sensitivities due to their overlapping roles and occupancies in an estuary. Thus, any environmental implications of differences in LULC within an estuary's watershed may be indicated by the observed counts of species that may have similar life histories, habitats, or feeding behaviors. The richness of all three FGs showed strongest associations with increases in estuarine wetland, which further supports the scientific understanding of the ecological benefits of estuarine wetlands (Barbier et al., 2011; Boesch & Turner, 1984; Reed et al., 2009). These benefits include water quality maintenance and wave attenuation, which have been shown to positively affect habitat suitability for fishes in other studies (e.g., Engle, 2011). Estuarine emergent and shrub wetlands also provide nursery habitat for many fish and other aquatic species (Boesch & Turner, 1984), which demonstrates further evidence supportive of the positive relationships observed in this study between estuarine wetlands and species richness. A similar relationship was observed between species richness and palustrine wetlands, which provide ecosystem services similar to those of estuarine wetlands but are particularly effective at regulating nitrogen inputs (Jordan et al., 2011), which helps mitigate eutrophication within estuaries. The negative relationship observed between shrimp species richness and estuaries with higher palustrine wetland influence may have ties to mitigated nutrient inputs and is worth exploring further.

The two anthropogenic land‐cover types, development and cultivated cropland, demonstrated mixed relationships between their levels of influence and species richness. The decline in expected species richness of all FGs to increases in developed land influence is consistent with the loss of ecosystem functions tied to increases in development (Lowe & Peterson, 2014; Peterson & Lowe, 2009; Short & Burdick, 1996). The greater magnitude in the decline of pelagic and forage finfish groups may be explained by the higher mobility of species in these groups compared with species within the shrimp group. In the cases of pelagic and forage finfish, movements may be influenced by seasonal changes or by temporary conditions within an estuary that are suboptimal, such as high freshwater inflow from the watershed. In contrast, shrimp have been shown to tolerate pollution from urbanization provided that structural habitat provisions are present (Ramírez et al., 2012). Cropland influence appeared to be associated positively with shrimp richness and negatively associated with forage finfish richness. The positive association of shrimp richness with cropland may stem from increases in primary production from increased nutrient loading due to agriculture, which could in turn result in more detrital loading, which is a valuable food source for shrimp (Chong & Sasekumar, 1981; Tiews et al., 1976). Of course, considering how shrimp associate with land use differently from forage finfish or pelagic species needs to be considered when determining conservation or management priorities. Prioritizing shrimp diversity may not need to incorporate as much palustrine wetland or forestland protections and may allow for more agricultural land use to remain within a watershed. Overall, while the results suggest that there may be ecological significance in the trends our models detected, there is no conclusive significance. At the regional scale, these trends are relevant, but as we approach the scale of individual estuaries, more context would be required to accurately model the system.

The models are limited in how they address land‐to‐sea processes, which may imply process uncertainty within the models. We chose to represent LULC influence on each estuary through just one mechanism, annual runoff, which could be a broadly relevant metric for comparing LULC and its water quality effects, given the large geographic area and diversity of the GCR. However, our models leave out all other aquatic processes that occur between land and the estuary, such as sediment transport and nutrient loading, which might explain more of the uncertainty that we see in our model results. This modeling approach is intended to have additional processes added to it as sufficient data to model said processes become available for the region of interest. The benefit of using a Bayesian framework in this instance is that it treats all unobserved quantities as random variables and can include many more components without much risk of overfitting.

Our modeling approach comprises several sources of uncertainty that need addressing. A primary source of uncertainty is our exclusion of all portions of each watershed that extend beyond the C‐CAP data coverage. For many estuaries, this means that headwater portions of the watersheds were not included in the analysis, which could undoubtedly influence our response variable (Miller et al., 2018). However, the focus of this study centered on modeling the association of estuarine biotic health with coastal and near‐coastal lands, which have more direct linkages to conditions within estuarine environments, generally making headwater LULC influence less relevant. The Mississippi River basin, excluded from this study, is an exception to the preceding statement because of the basin's anomalous size and extent of anthropogenic manipulation (e.g., channelization, diversion of flow, floodplain restrictions), with inputs from the basin resulting in hypoxic zones in the Northern Gulf of Mexico averaging 15,000 km^2^ since 1993 (Rabalais et al., 2002). We also excluded grassland and pasture from our study owing to issues with model convergence when we included them as covariates. Future work should attempt to reintroduce those variables into the models, as they could reveal more associations between land use and estuarine biotic health.

Other uncertainties surround our characterization of the water cycle and stem from not distinguishing where LULC classes are located in relation to the estuary. Our use of the curve number method to describe runoff from LULC classes is known to be a robust and elegant means of estimating runoff. However, this method's limited incorporation of the water cycle needs to be recognized because it does not consider groundwater recharge or evapotranspiration. Forests and wetlands have a great capacity to store water within a landscape and contribute to increases in groundwater recharge (Adane et al., 2018) and evapotranspiration rates (Roberts, 1983) on the landscapes in which they occur. Because our model does not account for these aspects of the water cycle, some influences of runoff variation are overlooked. Future iterations of this model should try to incorporate more aspects of the water cycle to improve estimates of runoff. Another source of uncertainty comes from not considering the orientation of LULC classes in terms of their configurations with respect to hydrologic features. Our results should not be invalidated by this factor, but it should limit our ability to further distinguish conservation value of sites within the same watershed. The next phase of this research is to look into the degree of hydrologic connectivity between LULC classes and each estuary, which should provide more locational context to the influence of each LULC class on estuarine biotic health.

Our study also did not examine heterogeneity in detection probability, which in some cases may indicate that a parameter is correlated with the probability of observing a species that is present. Despite this, LULC parameters should be independent of any factors that may impact detection probability from trawl samples. This complication could potentially yield inaccurate results that could over‐ or underestimate species presence and confound relationships that we infer between species richness and environmental covariates (McNew & Handel, 2015). The main factors that could influence detection probability of different taxa are the personnel experience and protocols associated with different trawl programs, which we account for by allowing the intercept to vary by trawl program in our models. Different detection probabilities, if true, could bias results.

Our models also did not incorporate fishing pressure as a covariate, which could further explain variation, especially for species that are recreationally or commercially important. Despite the potential importance of fishing pressure, the resolution and extent of potential data sources precluded the possibility of distinguishing fishing pressure among different estuaries. For instance, the Marine Recreational Fisheries Statistics Survey (MRIP), produced by NOAA, reports harvest of individual species by state at the highest resolution, which could represent statewide fishing pressure, but would not allow one to explore fishing pressure by estuary. More spatially descriptive fisheries data, where available, would be useful to include to perhaps explain some uncertainty in modeling land‐to‐sea processes.

## CONCLUSION

These models can provide a quantitative basis for assigning offsite values to lands for conservation potential for benefitting estuarine health and could be used to inform conservation planning along the Gulf Coast of the United States. With the model results, someone could consider the value of the natural land cover that is found within an area of interest (AOI) based on the expected species richness that pertains to the estuary the AOI drains to. A subsequent study will investigate how to construct a value model of land conservation potential for benefitting estuarine health that incorporates model results. In addition to modeled associations between LULC and expected species richness, a land conservation value model should also consider the hydrologic connectivity of the AOI to an estuary and the threat of conversion or loss of land cover. Conversion may happen due to development, which could be estimated using predicted urbanization scenario models (e.g., SLEUTH models; Clarke, 2008; Terando et al., 2014) and by extreme weather events and sea‐level rise, which could increase erosion and inundation, along with subsequent loss of ecosystem functions (Borchert et al., 2018). Remaining aware of the sources of land loss or conversion is vital in considering areas for effective conservation.

The simplicity of the proposed model is partially motivated by a desire to provide a transferable model that can make use of readily available georeferenced data. Despite the assumptions inherent in the modeling approach, the methodology appears useful for conveying quantitative linkages between LULC and estuarine biotic health. We encourage the use of the Bayesian hierarchical approach in other ecoregions for further validation, because the estuaries along the Gulf Coast Region of the United States are not necessarily reflective of estuaries found in other regions. Particular to the Bayesian approach, one can include more parameters without running as much risk of overfitting as one would in a frequentist approach. This allows for more flexibility in incorporating ecological processes that are relevant to land–sea interactions. An additional advantage of the Bayesian approach is that it provides covariate estimates as a probability distribution, which can be directly interpreted as the likelihood that a parameter will have a nonzero effect on a response. Furthermore, users of the Bayesian approach could take advantage of informing prior distributions to improve the convergence of model estimates. However, much caution is advised in using informative priors because misguided prior knowledge can result in overfitting. Even in the absence of data that could clarify some uncertainty within the process (e.g., flushing time of estuary, pH, depth, sampling personnel), the hierarchical approach we implemented can help account for unmeasured factors through grouping data by estuary and sampling program (Gelman & Hill, 2006). Future improvements to this approach could take advantage of the flexibility afforded to Bayesian hierarchical models by adding more complexity and dimensionality to the modeled process without reducing the interpretability of individual model components.

## CONFLICT OF INTEREST

The authors declare no conflict of interest.

## Supporting information


Appendix S1

Data S1
