# Supplementary material for: Considering the influence of land use/land cover on estuarine biotic richness with Bayesian hierarchical models
Source: Ecol Appl. 2022 Jul 14;32(7):e2675. doi: 10.1002/eap.2675 (PMC9786285; doi:10.1002/eap.2675)
Supplement: Supplementary file 1 — Appendix S1 [file EAP-32-e2675-s002.pdf]

Supporting Information. Shamaskin, Andrew Challen, Sandra B. Correa, Garrett M. Street, Anna C. Linhoss, and Kristine O. Evans. 2022. Considering the influence of land-use/land cover on estuarine biotic richness with Bayesian hierarchical models. *Ecological Applications.*

Appendix S1.

This appendix provides supplementary information about the trawl dataset, as well as more model results including cross-correlation tables, variances of group or random effects, and effect sizes for fixed parameters.

Table S1. Summary of trawl samples comprised for study from Florida (FL), Alabama (AL), Mississippi (MS), Louisiana (LA), Texas (TX), Environmental Monitoring and Assessment Program (EMAP), and the National Coastal Assessment (NCA).

Trawl Program	# of trawls	Dates of sampling
FL	9580	1991–2005
AL	2620	1991–2006
MS	708	1991–2005
LA	23,580	1991–2007
TX	31,870	1991–2009
EMAP	418	1991–1994
NCA	795	2000–2004

Table S2. Cross-correlation tables for all parameter estimates of fixed-effects within the Bayesian hierarchical model for the pelagic group.

	Intercept	Salinity	Temperature	Developed	Barren	Palustrine Wetland	Estuarine Wetland	Cultivated Cropland
Salinity	-0.029							
Temperature	-0.057	0.028						
Developed	0.001	-0.015	-0.002					
Barren	0.003	0.007	0.004	-0.626				
Palustrine Wetland	-0.030	-0.003	0.001	-0.141	0.211			
Estuarine Wetland	0.001	0.008	0.002	-0.334	0.240	-0.315		
Cultivated Cropland	0.020	0.002	0.005	-0.074	-0.140	-0.225	0.220	
Forest	0.002	-0.001	-0.006	0.052	-0.317	-0.422	0.361	0.151

Table S3. Cross-correlation tables for all parameter estimates of fixed-effects within the Bayesian hierarchical model for the forage finfish group.

	Intercept	Salinity	Temperature	Developed	Barren	Palustrine Wetland	Estuarine Wetland	Cultivated Cropland
Salinity	0.009							
Temperature	-0.021	-0.023						
Developed	-0.002	0.007	0.002					
Barren	0.002	-0.014	-0.005	-0.647				
Palustrine Wetland	-0.014	-0.001	0.006	-0.114	0.174			
Estuarine Wetland	0.006	-0.005	-0.006	-0.367	0.299	-0.371		
Cultivated Cropland	0.007	0.005	-0.005	-0.019	-0.178	-0.198	0.234	
Forest	0.005	0.003	-0.001	0.123	-0.361	-0.405	0.297	0.145

Table S4. Cross-correlation tables for all parameter estimates of fixed-effects within the Bayesian hierarchical model for the shrimp group.

	Intercept	Salinity	Temperature	Developed	Barren	Palustrine Wetland	Estuarine Wetland	Cultivated Cropland
Salinity	-0.027							
Temperature	0.041	-0.014						
Developed	-0.005	0.008	-0.003					
Barren	0.005	-0.003	0.005	-0.665				
Palustrine Wetland	-0.015	-0.010	-0.004	-0.197	0.200			
Estuarine Wetland	0.016	0.005	-0.003	-0.325	0.301	-0.369		
Cultivated Cropland	0.016	0.004	-0.010	0.081	-0.219	-0.337	0.300	
Forest	0.002	-0.001	0.001	0.149	-0.331	-0.404	0.237	0.137

Table S5. Variance of random intercepts and slopes for each functional group's model.

Model	Group	Name	Variance	Standard Deviation
Pelagic	Estuary	Intercept	0.670	0.819
		Salinity	0.288	0.537
		Temperature	0.426	0.653
	Trawl Program	Intercept	0.478	0.691
		Salinity	0.126	0.354
		Temperature	0.521	0.722
Forage Finfish	Estuary	Intercept	0.117	0.342
		Salinity	0.034	0.184
		Temperature	0.015	0.121
	Trawl Program	Intercept	0.090	0.300
		Salinity	0.003	0.052
		Temperature	0.007	0.083
Shrimp	Estuary	Intercept	0.184	0.429
		Salinity	0.042	0.205
		Temperature	0.036	0.190
	Trawl Program	Intercept	0.462	0.680
		Salinity	0.011	0.103
		Temperature	0.006	0.080

Table S6. Effect size of land-use/land-cover covariates for each functional group's model, reported as Cohen's d.

Model	Parameter	Effect Size (Cohen's d)
Pelagic	Developed	-0.101
	Barren	0.027
	Palustrine Wetland	-0.497
	Estuarine Wetland	0.056
	Cultivated Cropland	0.362
	Forest	-0.036
Forage Finfish	Developed	-0.180
	Barren	0.030
	Palustrine Wetland	0.379
	Estuarine Wetland	0.175
	Cultivated Cropland	-0.205
	Forest	0.390
Shrimp	Developed	-0.048
	Barren	-0.152
	Palustrine Wetland	-0.095
	Estuarine Wetland	-0.002
	Cultivated Cropland	0.260
	Forest	0.417
