# Supplementary material for: Considering the influence of land use/land cover on estuarine biotic richness with Bayesian hierarchical models
Source: Ecol Appl. 2022 Jul 14;32(7):e2675. doi: 10.1002/eap.2675 (PMC9786285; doi:10.1002/eap.2675)
Supplement: Supplementary file 2 — Data S1 [file EAP-32-e2675-s001.zip › DataS1/~WRL2563.tmp]

Appendix S1. Supplementary information from Bayesian hierarchical models

Pelagic model

Cross correlation tables

Variance of random intercepts and slopes

Effect size of land-use/land-cover coefficients

Forage Finfish model

Cross correlation tables

Variance of random intercepts and slopes

Effect size of land-use/land-cover coefficients

Shrimp model

Cross correlation tables

Variance of random intercepts and slopes

Effect size of land-use/land-cover coefficients
