# Supplementary material for: Considering the influence of land use/land cover on estuarine biotic richness with Bayesian hierarchical models
Source: Ecol Appl. 2022 Jul 14;32(7):e2675. doi: 10.1002/eap.2675 (PMC9786285; doi:10.1002/eap.2675)
Supplement: Supplementary file 2 — Data S1 [file EAP-32-e2675-s001.zip › MetadataS1.pdf]

Supporting Information. Shamaskin, Andrew Challen, Sandra B. Correa, Garrett M. Street, Anna C. Linhoss, and Kristine O. Evans. 2022. Considering the influence of land-use/land cover on estuarine biotic richness with Bayesian hierarchical models. *Ecological Applications.*

Metadata S1.

Contents:

This document describes the associated .zip file 'DataS1' which contains the following sub-folders and files:

README.txt, which states the manuscript title, authors, and journal.

Data:

1. trawl_estuary_data.csv, the dataset of trawl samples and estuarine-specific land-use/land cover (LULC) data.
2. trawl_estuary_holdout.csv, the dataset of trawl samples and estuarine-specific land-use/land cover (LULC) data not used in model fitting.

R:

1. Bayesian_Hierarchical_models.R, the R script that runs Bayesian hierarchical models for each of the functional groups (i.e. pelagic, forage finfish, and shrimp) assessed within the manuscript.

2. Posterior_Predictive_Checks.R, the R script that runs posterior predictive checks on each of the Bayesian hierarchical models within the manuscript.

JAGS:

1. Pelagic_Model.txt, the JAGS script that contains the Bayesian hierarchical model performed for the pelagic functional group.

2. Forage_Finfish_Model.txt, the JAGS script that contains the Bayesian hierarchical model performed for the forage finfish functional group.

3. Shrimp_Model.txt, the JAGS script that contains the Bayesian hierarchical model performed for the shrimp functional group.

Data Description:

Within 'trawl_estuary_data.csv' and 'trawl_estuary_holdout.csv' the following variables along with their descriptions are described below. Columns 16-21 utilized C-CAP land classes from 2001 (Office for Coastal Management 2019).

Estuary: Name of the estuary where the trawl sample took place.
NFHPSample: Sample identification number from the National Fish Habitat Partnership.
NFHPSite: The sample's site identification code from the National Fish Habitat Partnership.
Program: The trawl-sampling program that conducted the given sample.
State: The state where the sample occurred.
Latitude: Latitude coordinates of the trawl sample's midpoint, in decimal degrees.
Longitude: Longitude coordinates of the trawl sample's midpoint, in decimal degrees.
YYYY: 4-digit year of sample.
MM: 2-digit month of sample.
DD: 2-digit day of sample.
Temperature_Mean: Average water temperature during the sample, in °C.
Salinity_Mean: Average water salinity during the sample, in ppt.
Spp_Count_Pelagic: The species count of aquatic organisms observed within the trawl sample classified as pelagic.
Spp_Count_Shrimp: The species count of aquatic organisms observed within the trawl sample classified as shrimp.
Spp_Count_Forage_Finfish: The species count of aquatic organisms observed within the trawl sample classified as forage finfish.
Developed: Runoff volume per the combination of C-CAP developed classes within each estuary's watershed, normalized by watershed area $(m^3/_{km^2})$.
Palustrine_Wetland: Runoff volume per the combination of C-CAP palustrine wetland classes within each estuary's watershed, normalized by watershed area $(m^3/_{km^2})$.
Estuarine_Wetland: Runoff volume per the combination of C-CAP estuarine wetland classes within each estuary's watershed, normalized by watershed area $(m^3/_{km^2})$.
Barren: Runoff volume per C-CAP's barren class within each estuary's watershed, normalized by watershed area $(m^3/_{km^2})$.
Cultivated_Cropland: Runoff volume per C-CAP's cultivated cropland class within each estuary's watershed, normalized by watershed area $(m^3/_{km^2})$.
Forest: Runoff volume per the combination of C-CAP forest classes within each estuary's watershed, normalized by watershed area $(m^3/_{km^2})$.

Code Descriptions:

R scripts were created with R 3.6.0 (R Core Team 2019) and JAGS scripts were created with JAGS 4.3.0 (Plummer 2017). All R and JAGS scripts are provided within an R project titled 'DataS1' and thus there is no need to adjust the code to change the working directory. The codebase from the 5 scripts (i.e. Bayesian_Hierarchical_models.R, Posterior_Predictive_Checks.R, Pelagic_Model.txt, Forage_Finfish_Model.txt, and Shrimp_Model.txt) can all be called by just running Bayesian_Hierarchical_models.R. For each of the 3 models within Bayesian_Hierarchical_models.R (i.e. Bayes_Pelagic,

Bayes_Forage_Finfish, Bayes_Shrimp), a set of initial values was specified with the objects 'initslist_Pelagic', 'initslist_ForageFinfish', and 'initslist_Shrimp'. After the 3 models are run, the posterior predictive checks are called through the source() function.

The Bayesian models were run with parallel processing in order to optimize runtimes, as specified within the autorun.jags() function using the arguments method='parallel' and n.sims=4. If the computer being used to run the models has a different number of processors than what the code in 'Bayesian_Hierarchical_models.R' specifies, the 'method' and 'n.sims' arguments will need to be adjusted to reflect the capabilities of the computer being used.

Literature Cited:

Office for Coastal Management. 2019. NOAA's Coastal Change Analysis Program (2001) Regional Land Cover Data – Coastal United States, https://inport.nmfs.noaa.gov/inport/item/48330.

Plummer, M. 2017. *JAGS version 4.3.0 user manual [computer software manual]*. Retrieved from https://martynplummer.wordpress.com/2017/07/18/jags-4-3-0-is-released/

R Core Team. 2019. R: A language and environment for statistical computing. Vienna, Austria.
